# The Nucleolus: A Multiphase Condensate Balancing Ribosome Synthesis and Translational Capacity in Health, Aging and Ribosomopathies

**DOI:** 10.3390/cells8080869

**Published:** 2019-08-10

**Authors:** Carl C. Correll, Jiri Bartek, Miroslav Dundr

**Affiliations:** 1 Center for Proteomics and Molecular Therapeutics, Rosalind Franklin University of Medicine & Science, North Chicago, IL 60064, USA; 2 Danish Cancer Society Research Center, Genome Integrity Unit, DK-2100 Copenhagen, Denmark; 3 Division of Genome Biology, Department of Medical Biochemistry and Biophysics, Science for Life Laboratory, Karolinska Institute, SE-171 77 Stockholm, Sweden; 4 Center for Cancer Cell Biology Immunology and Infection, Rosalind Franklin University of Medicine & Science, North Chicago, IL 60064, USA

**Keywords:** genome instability, nucleolus, phase separation, pre-rRNA processing, rDNA genes, ribosome biogenesis, ribosomal genes, links to diseases

## Abstract

The nucleolus is the largest membrane-less structure in the eukaryotic nucleus. It is involved in the biogenesis of ribosomes, essential macromolecular machines responsible for synthesizing all proteins required by the cell. The assembly of ribosomes is evolutionarily conserved and is the most energy-consuming cellular process needed for cell growth, proliferation, and homeostasis. Despite the significance of this process, the intricate pathophysiological relationship between the nucleolus and protein synthesis has only recently begun to emerge. Here, we provide perspective on new principles governing nucleolar formation and the resulting multiphase organization driven by liquid-liquid phase separation. With recent advances in the structural analysis of ribosome formation, we highlight the current understanding of the step-wise assembly of pre-ribosomal subunits and the quality control required for proper function. Finally, we address how aging affects ribosome genesis and how genetic defects in ribosome formation cause ribosomopathies, complex diseases with a predisposition to cancer.

## 1. Introduction

The ribosome is fundamental to cellular function, serving as the site for protein synthesis. Ribosome production and protein translation are tightly coordinated to ensure that the number of ribosomes supports the demands for protein synthesis associated with cell growth and proliferation. In humans, biosynthesis of ribosomes depends on the coordinated activity of three RNA polymerases to transcribe four noncoding ribosomal RNAs (rRNAs), greater than 150 small nucleolar RNAs (snoRNAs), the genes encoding approximately 80 ribosomal proteins (r-proteins) and the >200 trans-acting assembly factors (AFs). The process is initiated by transcription of rDNA genes using the dedicated RNA polymerase (pol) I machinery. The resulting production of the 47S pre-rRNA precursor initiates nucleation of the nucleolus around the ribosomal genes within the cell nucleus. Trans-acting AFs coordinate stepwise and hierarchical modifications, processing and folding of the 47S pre-rRNA precursor, and its assembly with r-proteins. Ribosomal assembly begins in the nucleolus, migrates to the nucleoplasm and culminates after export to the cytoplasm, where final processing and assembly occur, and quality control ensures that only active ribosomal subunits are released. From this perspective, we focus on recent progress elucidating the role of liquid-liquid phase separation in the formation of the nucleolus, the functional multiphase sub-organization of the nucleolus, the epigenetic regulation of rDNA gene expression, and the structure and function of the pre-rRNA processing machinery. Lastly, we examine the pathophysiological links between ribosome biogenesis, the nucleolus, aging, and ribosomopathies.

## 2. Phase Separation and the Dynamic Nature of the Nucleolus

**Figure 1:**
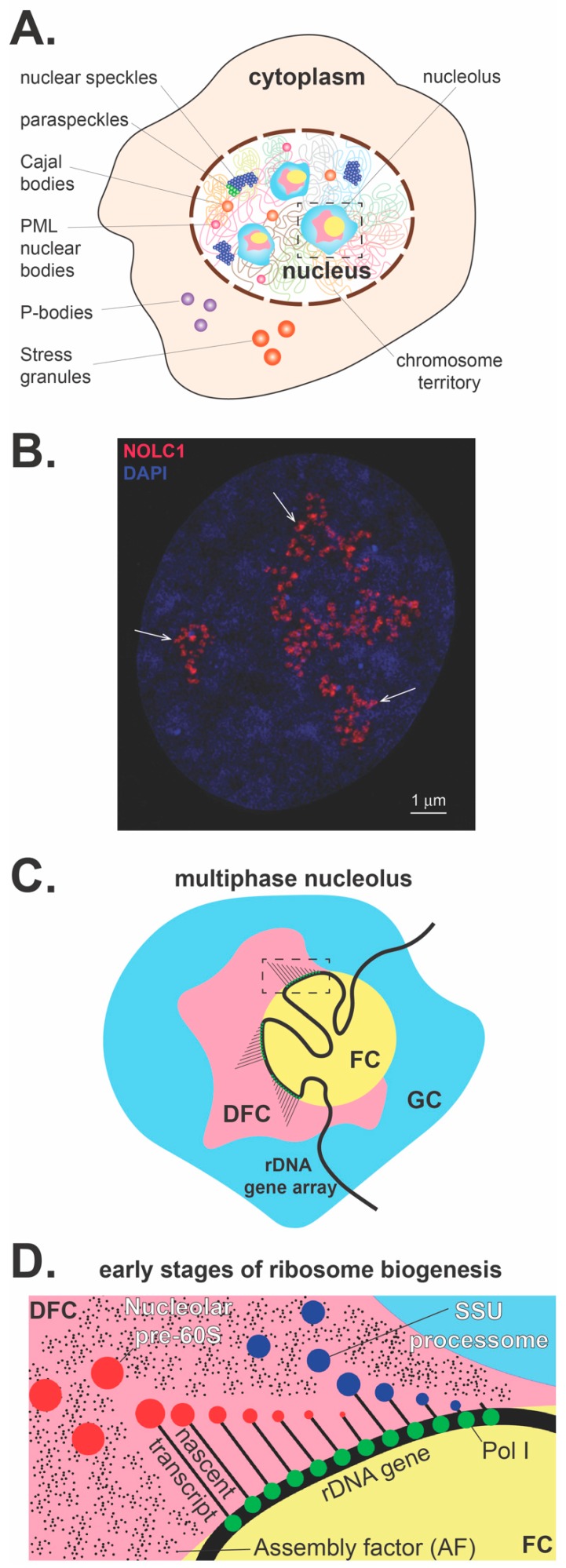
The nucleolus overview. (**A**) Within the cell there are a myriad of membrane-less subcompartments that include cytoplasmic processing bodies and stress granules. Within the nucleus, numerous subcompartments that include the most prominent membrane-less organelle, the nucleolus, nuclear speckles, paraspeckles, Cajal bodies, and PML nuclear bodies. (**B**) Nucleolar pre-rRNA processing sites visualized in a human U2OS cell. *NOLC1*, a nucleolar chaperone interacting with box C/D and box H/ACA small nucleolar ribonucleoproteins (snoRNPs), is detected by a specific antibody, co-stained with DAPI, and visualized by structured illumination microscopy. This nucleolar factor is primarily localized in regions of early pre-rRNA processing events, which correspond to the phase-separated subnucleolar phase corresponding to previously characterized dense fibrillar components (arrows, size bar 1 micron). (**C**) The enlarged nucleolus with three nucleolar subcompartments is depicted. The multiphase nucleolus is organized around rDNA gene arrays, which are anchored in the fibrillar centers (FCs). Transcriptionally active rDNA genes are located at the fibrillar center/dense fibrillar component (FC/DFC) interface. Nascent pre-rRNA transcripts are entering DFCs, where they undergo a series of processing, modification, and folding steps. Later, pre-rRNA processing and assembly steps of pre-ribosomal particles occur in the granular component at the periphery of the nucleolus (GC). (**D**) A detailed representation of the inset in panel C diagrams the co-transcriptional assembly with AFs and processing that releases the nucleolar assembled SSU processome and the LSU pre-ribosome within the DFC.

Numerous self-organizing membrane-less subcompartments, collectively referred to as biomolecular condensates (BMCs), exist in the cell nucleus and cytoplasm and share several key biophysical and material properties [1,2,3,4,5]. BMCs form dynamically as supramolecular assemblies within the cell to locally concentrate specific proteins and nucleic acids whose self-assembly and structure are linked to many essential cellular processes [6,7]. In the cytoplasm, BMCs include, as main examples, processing bodies (P-bodies), stress granules, Lewy bodies, germ granules and centrioles. Within the nucleus, BMCs include the nucleolus and other prominent nuclear domains, such as nuclear speckles, paraspeckles, Cajal bodies, and Promyelocytic leukemia (PML) nuclear bodies (Figure 1A). BMCs exhibit liquid-like properties and form via liquid-liquid phase separation of their molecular components [8,9,10]. During phase separation, the critical concentration of specific constituents (either proteins or RNAs) promotes the spontaneous demixing of molecules from a homogenous single phase into a concentrated phase that is physically distinct from the dilute phase, forming two phases that stably coexist. Thus, for example, these distinct phases can create a discrete liquid phase within an enveloping immiscible liquid phase, which is essentially a more concentrated core within a less concentrated liquid shell [1,11]. In this manner, phase separation can sequester components into specific phases, with many functional consequences yet to be fully explored. For example, sequestration could prevent biochemical reactions, as exhibited by how cytoplasmic stress granules sequester mRNA, to prevent translation in times of stress.

The site of rRNA synthesis, processing and early steps of pre-ribosome assembly is the nucleolus, which is the most prominent membrane-less nuclear structure, accounting for 20 to 25% of the total nuclear volume (Figure 1A,B). Remarkably, a growing human aneuploid HeLa cell (cervical carcinoma) synthesizes approximately 7500 ribosomal subunits every minute [12], which perhaps reflects the upper limit for the rate of ribosome production, given that these are cancer cells. To meet these demands, the viscous nature of the nucleolus is ideally suited to dynamically coordinate the exchange and interactions of hundreds of required components and factors needed to produce and export pre-ribosomal particles. The dynamic nature of phase-separated nucleolar organization locally concentrates components and RNA substrates into reaction centers, potentially to promote rapid ribosome production by changing reaction specificity and accelerating the rates of reaction and assembly.

**Figure 2:**
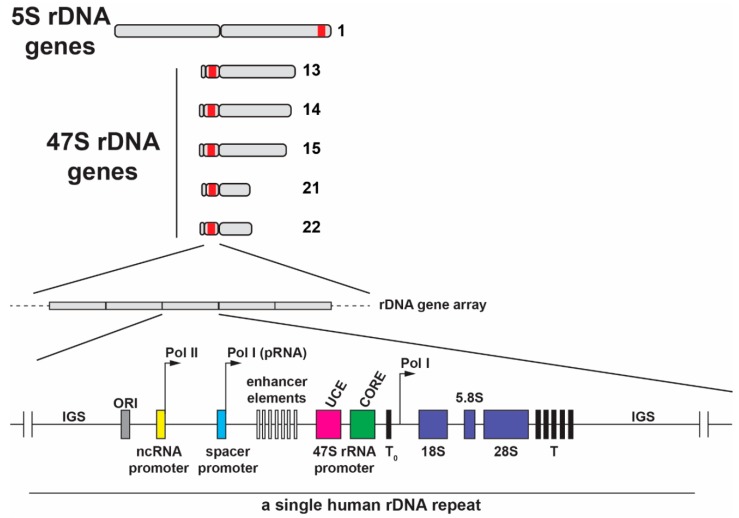
The location of the 5S rDNA gene array in the q arm of human chromosome 1 and the 47S rDNA gene repeats located in the short p arms of the five acrocentric human chromosomes. Shown below is a schematic of one human rDNA gene repeat, which is separated by intergenic spacers (IGSs). The human pre-rRNA coding region consists of 18S, 5.8S and 28S rRNA sequences and transcribed two ETS and two ITS spacers. RNA pol I transcription is initiated at the 47S core promoter and transcription ends at multiple downstream promoter terminator elements (T). A terminator-like sequence (T_0_) is located near the core promoter. IGSs also contain repetitive enhancer elements including the upstream control element (UCE), core promoter element, the origin of replication (ORI), and spacer promoters. Non-coding pRNA is transcribed by RNA pol I and it originates from the spacer promoter located in IGS. Additionally, several long non-coding RNAs located in IGS are transcribed by RNA pol II under stress conditions.

The nucleolus is formed by the on-going transcription of tandemly arranged conservative repeats of ribosomal RNA genes, which are designated nucleolar organizer regions (NORs) [13]. In human cells, NORs are situated in secondary constrictions between the centromeres and telomeres on the short p arms of the five acrocentric chromosomes 13, 14, 15, 21 and 22 (Figure 2). Transcriptionally active phase-separated rDNA arrays progressively coalesce to form between one and three large nucleoli in mammalian nuclei, which stably cluster in close spatial proximity to the NOR-bearing chromosome territories [14,15] (Figure 1A–C).

Specific coding or non-coding RNAs play a key role in the formation of morphologically and molecularly distinct BMCs [16,17]. These RNAs can provide a scaffold that attracts and retains specific RNA-binding proteins [18,19,20]. Formation of the nucleolus (and other nuclear BMCs) is driven, along with rDNA gene expression, by multiple transient low-affinity protein-protein interactions. These involve long stretches of intrinsically disordered regions (IDRs) of proteins consisting of low-complexity amino acid sequences with high conformational flexibility [3,21,22]. IDRs frequently increase an intra-BMC bonding network through their proximity to specific RNA-binding domains responsible for sequence-specific RNA recognition and binding [23]. These ribonucleoprotein (RNP) complexes initiate numerous intermolecular low-affinity interactions to form interactions with essential proteins that serve as multivalent stochastic hubs, transiently binding to dozens or even hundreds of distinct client proteins through weak binding affinities. Concentrated molecules within a phase-separated liquid condensate continuously exchange with the surrounding solution while the integrity of the condensate stably persists [24].

Notably, more than 80% of all RNA found in the growing mammalian cell is related to ribosomal RNA [25]. Based on the analysis of Miller chromatin spreads, there are between 100 and 120 nascent transcripts on each rDNA gene [26,27] with ~15,000 engaged RNA pol I molecules per Hela cell [28]. Thus, nucleoli exhibit the highest concentration of active genes in the nucleus. The high concentration of pre-rRNA transcripts is primarily responsible for the formation of a nucleolus.

Subsequent steps in the processing of pre-rRNA are followed by the assembly of pre-ribosome particles, which spatially coordinates the vectorial process of pre-ribosome biogenesis within the well-established multiple nucleolar substructures (Figure 1C). The three phase-separated layers correspond to the following well-known and functionally characterized nucleolar subcompartments: the fibrillar center (FC), the dense fibrillar component (DFC), and the granular component (GC) (Figure 1C) [3,29,30,31,32]. The nucleolar structure forms through differences in surface tension between the distinct liquid phases that arise from their respective macromolecular components [9]. Perhaps the physical environment of the three distinct liquid-like phases coordinates directed ribosomal processing with each phase characterized by particular inward and outward fluxes of specific proteins and RNA molecules. Although the specific role of actively transcribing rDNA genes in these coexisting phases has yet to be determined, it is speculated that the arrival of pre-rRNA recruits client proteins, which in turn drives phase separation. The spatial separation and distinct physical and compositional features of these three subnucleolar phases may optimize pre-rRNA processing, vectorial transport, and the step-wise, hierarchical assembly of pre-ribosomal subunits.

Transcription of rDNA is restricted to the centrally positioned FC/DFC interface. The early steps of pre-rRNA processing and base modifications (2′-*O*-methylation and pseudouridylation) occur in the DFC (Figure 1C,D). The 47S pre-rRNA precursor is polycistronic, containing the small subunit (SSU) rRNA (18S) and the large subunit (LSU) rRNAs (5.8S and 28S). Mature rRNA regions are separated by external transcribed spacers (5′-ETS and 3′-ETS) and internal transcribed spacers (ITS1 and ITS2), which are removed by endo- and exonuclease events in a stepwise manner. After the primary polycistronic pre-rRNA transcript assembles with some r-proteins and AFs, a single endonucleolytic cleavage step separates the rRNA precursors into pre-40S and pre-60S particles, which subsequently follow independent pathways of biogenesis and export [33].

The polycistronic rDNA containing rRNA from both ribosomal subunits ensures equimolar amounts of the small (18S) and large (5.8S and 28S) subunit rRNAs (excluding 5S rRNA) and dysfunction in stoichiometry contributes to some ribosomopathies (see below). In humans, 5S rRNA is encoded by a separate gene locus (1q42.13), which is arranged in a tandem array of 50-300 repeating units on the q arm of chromosome 1 (Figure 2) [34] and is independently transcribed by RNA pol III outside the nucleolus in the nucleoplasm. After export to the cytoplasm, the 5S rRNA requires assembly with ribosomal protein (r-protein) *RPL5*, after which it is imported back into the nucleolus. The number of 5S rDNA gene copies correlates with the 47S rDNA copy number to match the stoichiometric demands for each ribosomal subunit [35]. To further enable equimolar levels of 5S rRNA, RNA pol I activity co-regulates RNA pol III activity in response to environmental signals [36]. Equimolar production of each of the four mature rRNAs, which are indispensable structural and catalytic components of the ribosome, promotes efficient use of the vast cellular resources dedicated to ribosome synthesis.

Assembly of the pre-40S and pre-60S ribosomal subunits proceeds via independent pathways in the outer GC prior to movement to the nucleoplasm for additional assembly steps and in the case of the pre-60S particle additional remodeling and processing steps as well. Next, pre-40S and pre-60S subunits are independently exported to the cytoplasm for final processing, assembly, folding, and quality control. At this point, the final AFs dissociate, which signals activation of the mature 40S and 60S ribosomal subunits [37,38]. Finally, the largest known enzyme is made by joining the mature 60S subunit with an mRNA and charged initiator tRNA bound to the 40S subunit to form the mature translationally active 80S ribosome. At maturation in human cells, the small 40S subunit contains 33 r-proteins assembled around the 18S rRNA, while the large 60S subunit is composed of three different rRNAs (28S, 5.8S, and 5S) associated with 47 r-proteins [39,40].

The outer GC is also used as a limited buffer to temporarily sequester misfolded nuclear proteins that accumulated during stress conditions [41]. Storage of misfolded proteins in this way prevents irreversible aggregation that would impair nuclear function. When stress abates, these proteins can become substrates for Hsp70-assisted refolding.

## 3. Organization of Ribosomal Genes

NORs are located on the five acrocentric chromosomes: 13, 14, 15, 21, and 22 [42,43] (Figure 2). Previously, the rDNA gene sequence and copy number were thought to be highly conserved, in part due to a difficulty in sequencing these repetitive gene sequences. Recent progress in next generation sequencing this uncharacterized “dark matter” of the genome has revealed unexpected variation. The human NORs range in size from one repeat (40 kb) to >130 repeats (6 Mb) and consist of up to 400 repeats of an rDNA gene unit per diploid genome [43]. Moreover, there is nucleotide variation in the rDNA genes, which results in tissue-specific allelic variation in ribosomes [44] that may play a role in ribosomopathies (see below). The rDNA gene arrays are unevenly located on the short p arms in the secondary constrictions between centromeres and telomeres [45,46]. This topological arrangement positions them sufficiently far from the RNA pol II-driven protein-encoding genes that are regulated and transcribed outside the nucleolar body. Approximately 70% of human rDNA repeat units are organized in a canonical head-to-tail arrangement, with the remainder forming non-canonical, head-to-head arrangements [47]. Each rDNA repeat unit is ~43 kb in length with 13.3 kb transcribed as a single 47S polycistronic non-coding pre-rRNA transcript. The remaining ~30 kb forms a non-coding intergenic spacer between rDNA genes and contains regulatory elements such as gene and spacer promoters, repetitive enhancer elements, origins of DNA replication, transcription termination sites and several transcribed non-coding RNAs [48]. The short p arms of human NOR-bearing chromosomes have not yet been fully sequenced, although characterization of their genomic context and the NOR regulatory elements on the centromeric and telomeric sides of the rDNA gene arrays has begun (the Telomere-to-Telomere (T2T) consortium to generate the first complete assembly of a human genome) [43]. Frequently, the nucleolus is associated with specific chromatin clusters (nucleolus-associated chromatin domains, NADs) that are primarily heterochromatin and correlate with late-replicating loci. These comprise sub-telomeric regions, transposable elements (TEs), and largely inactive protein-coding genes [46,49,50]. Overall, identification of NADs reveals an active role of the nucleolus in the spatial organization of chromatin within a nucleus.

Ribosome biogenesis and protein translation must be well coordinated since their functional interplay is paramount for cell growth, proliferation, differentiation, development, and homeostatic tissue renewal and function. It has been established that rDNA genes exist in three states in mammalian cells: stably silent, inactive, and active. Approximately half of the rDNA genes are transcriptionally active at any given time, with the remainder permanently silenced. Stably silent genes are in compact heterochromatin structures, which are refractive to transcription due to extensive CpG methylation. This characteristic distinguishes them from the remaining euchromatin repeats [51]. Stably silent rDNA genes stay constitutively repressed and do not become transcriptionally active in cells, even during stages of high proliferation [52,53]. Active and inactive rDNA genes are in an open euchromatin conformation, which can be dynamically turned on or off for transcription. Typically, active rDNA genes are not methylated at CpG sites and are devoid of histones, thereby promoting accessibility to DNA [54]. Using a ChIP-Seq approach, Herdman and colleagues revealed that one exception is the Enhancer Boundary Complex, which is upstream of the spacer promoter enhancer repeats in IGS (Figure 2). It is associated with histones that can be dynamically modified to enhance or repress transcription and acts as the key entry point for the remodeling complexes that activate rDNA transcription [55]. In contrast, when the downregulation of global rRNA synthesis is necessary, rDNA genes adopt a chromatin structure that includes histone modifications associated with transcriptional repression (i.e., H3K9me3, H4K20me) but not CpG methylation, as the former modifications are readily reversible [51].

There are two ways to control rRNA synthesis: either 1) modulating the rate of transcription on each gene in an euchromatic state; or 2) adjusting the number of active genes. The first option involves regulating rDNA transcription by the post-transcriptional modulation of transcription factor activities (e.g., phosphorylation or acetylation) [56]. Upregulation occurs in response to external mitogenic signals that promote cell growth and proliferation, whereas downregulation occurs under conditions that impair cellular metabolism, such as nutrient depletion, genotoxicity, and oxidative stress. During pathological conditions such as cancer, the number of active rDNA genes will vary considerably among diverse cell types [57,58]. The second option involves long-term changes to the level of rDNA transcription reduce the number of active genes. This occurs predominantly by reduction in transcription factor upstream binding factor (UBF) levels and UBF loading on the rDNA genes [59] and the epigenetic condensation of rDNA genes [60,61].

### How Non-Coding RNA Maintains Heterochromatin by rDNA Silencing

The silencing of rDNA genes is regulated by upstream sequences present in the intergenic spacer region between rDNA genes (Figure 2). Transcription by RNA pol I of a non-coding 2-kb long intergenic spacer rRNA (IGS-rRNA) located 2-kb upstream of the main rDNA promoter is processed by the RNA helicase DHX9. The resulting mixture of 250–300 nucleotide RNAs is designated promoter-associated RNAs (pRNAs) [62,63]. pRNA folding into a stem-loop structure is a prerequisite for assembly with the repressor TTF1-interacting protein-5 (TIP5) in the nucleolus. This RNP associates with the Transcription Termination Factor 1 (TTF1) to guide and recruit the nucleolar repressive complex (NoRC) to the rDNA promoter. NoRC contains TIP5 and the DNA-dependent ATPase SNF2H (*SMARCA5*) and interacts with DNA methyltransferases (DNMTs) [64,65,66]. NoRC recruitment to the rDNA promoters leads to the accumulation of repressive marks, including CpG hypermethylation, hypoacetylation of histone H4, and H3K9me2/3 methylation, which silence rDNA genes and establish a condensed heterochromatic state [64]. Loss of CpG methylation by inactivation of DNMT leads to a loss of heterochromatin structure with a corresponding reactivation of silenced rDNA genes, and results in defects in pre-rRNA processing and cell proliferation [67]. pRNA is transcribed from hypomethylated rDNA genes during mid to late S phase and acts in trans to transcriptionally repress rDNA genes during mitotic division from one generation to the next because CpG methylation is inherited [63,68]. Recent evidence suggests that silencing rDNA genes is not limited to ribosome biogenesis but is also important in maintaining genome stability (reviewed in [69]).

## 4. Ribosomal Genes and Genomic Instability

The highly repetitive and heavily transcribed rDNA sequences are considered very fragile genomic sites [70]. They are frequent targets for unwanted homologous recombination events, causing genome instability. If not properly repaired uncontrolled recombination within the same or between different rDNA clusters may cause the translocation of chromosome arms, or insertions or deletions of repetitive sequences. Changes in rDNA repeat numbers from aberrant recombination can cause genomic instability within rDNA repeats and lead to deleterious effects, such as increased sensitivity to cytotoxic DNA, double-strand breaks (DSB) or impaired repair of DNA [71] (rDNA repair has been recently reviewed in [70]). The intrinsically unstable rDNA is further destabilized in cells defective in genome maintenance factors, thereby further emphasizing the urgent need for continued surveillance of nucleolar rDNA [72]. The evolutionarily conserved fraction of stably silenced rDNA genes in heterochromatin structure promotes genome stability because compact heterochromatic rDNA genes are less accessible to damage and to the DNA recombination machinery [73]. In contrast, active and inactive rDNA repetitive sequences undergo frequent deleterious recombination, giving the NORs the highest copy number variation in the genome [58]. Given the highly recombinogenic nature of rDNA and its potentially serious threat to genomic integrity, it is perhaps not surprising that nucleolar transcription is promptly silenced by the cellular DNA damage response machinery upon genotoxic stress, possibly to minimize the threats of deleterious recombinations of rDNA and facilitate proper repair of the rDNA [74,75,76,77].

In response to nucleolar DSBs, the ATM kinase becomes activated to signal repression of nucleolar transcription, nucleolar segregation and translocation of rDNA into so-called nucleolar caps at the nucleolar periphery [74,75,77]. Such restructuring of the nucleolus and localization of rDNA into nucleolar caps may provide a mechanism to separate rDNA genes localized on different chromosomes to avoid inter-chromosomal recombination events in response to DNA damage [43]. In agreement with this concept, the homologous recombination repair factors were shown to be recruited to the nucleolar caps formed at the nucleolar periphery after DNA damage induction [74,77,78]. Further attesting to the unique vulnerability, and careful surveillance of rDNA genes, the sensing, processing, and repair of the most deleterious DNA lesions, DSBs, have recently been shown to proceed via a unique mechanism distinct from DSB processing and repair in the chromatin outside the nucleoli [79]. Thus, the DNA DSBs in rRNA genes that are not immediately repaired by the non-homologous end joining (NHEJ) mechanism in the center of nucleoli are known to activate the ATM kinase, repress rRNA transcription and induce nucleolar cap formation [70,79]. Recent studies have shown that when such persistent rDNA DSBs are left unrepaired by NHEJ, they are detected by the ATM-phosphorylated nucleolar protein TCOF1 in combination with the MRE11-RAD50-NBS1 (MRN) complex. The DNA damage response kinase ATR then operates downstream of the ATM-TCOF1-MRN pathway to fully suppress rRNA transcription and translocate the unrepaired DSBs to nucleolar caps accessible by the error-free homologous recombination pathway, the components of which are kept outside the nucleoli to avoid the hazardous recombination of rDNA in the transcriptionally active nucleolar center. Interference with this specialized nucleolar DNA damage response mechanism undermines genomic stability and cell viability, documenting its genome-protective role [79].

## 5. Structural Insights on Pre-rRNA Processing from Cryo-Electron Microscopy

Due to the absence of 3D structural work on mammalian ribosomes, we will review the cryo-EM structures of intermediates in yeast ribosome biogenesis, which remains the model organism for human ribosome biogenesis. The structure of the mature yeast ribosome [80] highlighted several fundamental questions regarding ribosome biogenesis. For example, how does rRNA fold correctly to create active sites of the mature 80S ribosome such as the decoding center, which ensures that the anticodon of the tRNA complements the codon of the mRNA, and the peptidyl transferase center, which catalyzes peptide bond formation? Moreover, what roles do AFs and the external and internal transcribed spacers of pre-rRNA (5′ETS, 3′ETS, ITS1 and ITS2, respectively) play in this folding process? The recently reported cryo-EM structures of various intermediates between pre-rRNA transcription and the mature ribosome from yeast begin to address these questions (see [38] for a comprehensive review). The folding of rRNA is a daunting task because the rRNAs are so large (18S is 1798 nucleotides, 25S is 3392 nucleotides, and 5.8S RNA is 158 nucleotides in yeast). In addition, RNA can be trapped in stable nonfunctional forms. Unlike the relatively weak forces that stabilize the folding of protein structures (e.g., helicases and beta-sheets), about half of the folded rRNA structure is composed of the relatively more stable A-form helices. An incorrect helical structure that forms is stable and thus likely to trap the rRNA in a nonfunctional form. Another mystery is why so many AFs are needed. A few enzymes are needed to remove the ETSs and ITSs, to post-translationally modify the rRNA bases and sugars and to remodel the rRNA (e.g., dislodge the U3 snoRNP by unpairing the U3-pre-rRNA hybridization). It is less clear why over 200 AFs are needed and what roles they play. Lastly, why do we need ETSs and ITSs, which comprise about half of the primary rRNA transcript? All RNA polymerases are known to have higher error rates at both termini (5′ and 3′-ends) so external spacers are needed, but what is the necessity of hundreds of nucleotides in length and why are there internal spacers? These spacers are found throughout evolution. While their sequences vary, they all appear able to fold into a series of helical stem structures. Why is this the case?

Molecular snapshots of intermediates along the maturation pathway shed light on the many roles of AFs [81,82,83,84,85,86,87,88,89,90,91,92]. Some AFs transiently block key events by preventing rRNA misfolding, premature formation of mature structures, or premature binding of AFs (required at a subsequent stage). As the subunit structures begin to resemble their mature forms, other AFs bind to act as molecular mimics of translation factors or substrates (e.g., tRNA or mRNA), thereby blocking factor and/or substrate binding to keep immature particles translationally silent. Another group of AFs act as structural hubs that reach out across rRNA domains and provide binding sites for additional AFs (e.g., Mpp10 and Erb1 during SSU and LSU maturation, respectively [85,93]). Still others chaperone rRNA folding. During rRNA folding some domains fold into subdomain structures resembling their mature counterparts during early intermediates (e.g., subdomains near the 5′ end of 18S and 25S during SSU and LSU maturation, respectively), while others start as disordered entities and form in a step-wise hierarchical fashion (e.g., domains III, IV and V of 25S). Once active sites are formed, quality control mechanisms mediate dissociation of the final AFs to signal that maturation is complete. Interestingly, the folding and assembly of r-proteins and AFs occur co-transcriptionally.

**Figure 3:**
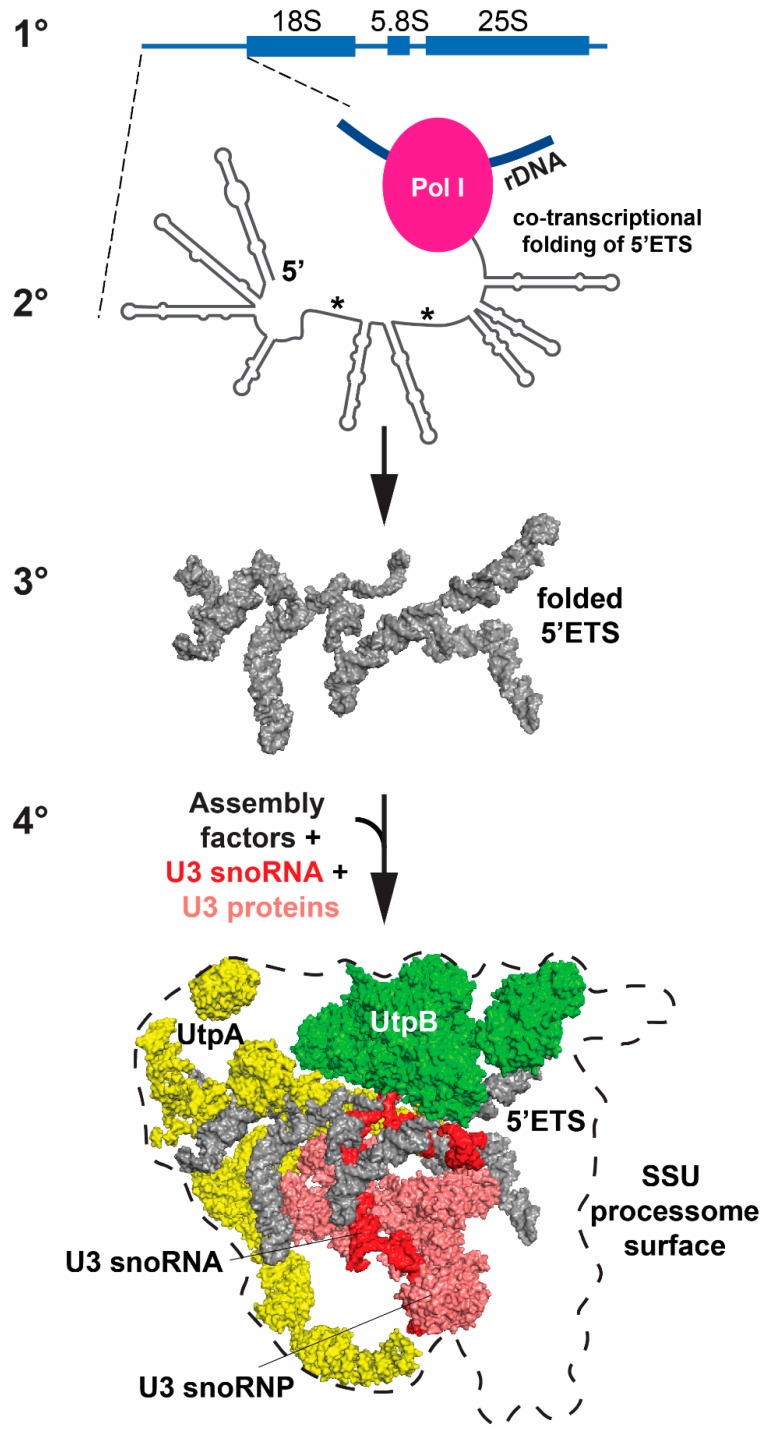
Hierarchy of RNA folding: the primary (1°) structure of the pre-rRNA sequence is represented schematically in linear fashion; the secondary (2°) structure shows the stem-loop of the 5′ETS, which folds co-transcriptionally; the tertiary (3°) structure shows folding of the stem-loop structure of the 5′ETS; and the quaternary (4°) structure shows how the folded 5′ETS acts as a landing pad to recruit protein assemblies (UtpA [yellow] and UtpB [green]) and the U3 snoRNP (RNA [red] and protein part [salmon]) (PDB ID 5WYK). The asterisks identify sites where the 5′ETS hybridizes with the U3 snoRNA (see Figure 4A, left panel).

**Figure 4:**
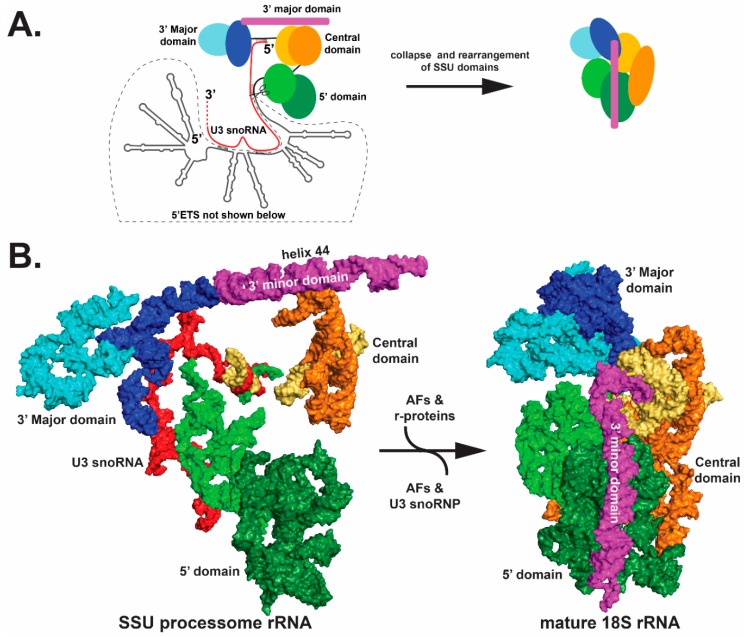
Collapse of the pre-ribosome SSU processome (left) into the mature ribosome (right). Only the rRNA components are shown for clarity. (**A**) A schematic view of the SSU processome (left) and the mature 18S (right) is shown. The 5′ domain, the central domain and the 3′ Major and minor domains initially fold as subdomains, which are colored in different shades of green, orange-yellow, blue and magenta, respectively. The rRNA subdomains undergo structural rearrangements between the left and right panels, as indicated by shape changes. Most of the domains undergo significant movement — particularly the 3′ minor domain and the central domains. (**B**) cryo-EM structures of the schematics are shown in panel (**A**); however, the 5′ETS is not shown in the left panel of (**B**). For clarity, only the rRNA is shown (PDB ID 5WYK). Orientation of the structure is the same as shown in Figure 3.

Structural biology has also provided insights into the role of external and internal transcribed spacers. The 5′ external transcribed spacer (5′-ETS) illustrates a role for the spacer elements [81,83,86,90]. As the nascent 47S transcript emerges from RNA pol I machinery, RNA stem-loop structures of the 5′ETS form and fold to provide a platform for hierarchical assembly of AFs, r-proteins and folding of the four SSU domains (Figure 3). As these structures form co-transcriptionally, they create landing sites to promote ordered assembly of a series of AF complexes, including the U3 small nucleolar RNP (snoRNP), a core component of the SSU processome (Figure 3). The U3 snoRNA base pairs with nucleotides in the pre-rRNA at several sites: the 5′ETS; the 5′ end of 18S (the 5′ domain); and nucleotides between the central and 3′ Major domains (Figure 4A). U3 snoRNP docking, together with bound AFs, splay open the four SSU domains. This enlarged structure blocks formation of the decoding center and a central and universal tertiary structural contact (the central pseudoknot) in the SSU. If a key functional feature of the 5′ ETS is the ability to form stem-loop structures that provide platforms for AFs to assemble, then sequences could vary as long as pairing is maintained. Perhaps this is the reason these transcribed spacers can tolerate so much sequence variation.

RNA helicases are expected to orchestrate many RNA-RNA structural rearrangements, including snoRNP dissociation. The RNA helicase Dhr1 [94] dislodges the U3 snoRNP and the excised 5′ETS along with associated AFs by a regulatory mechanism that is not yet understood. This exodus of factors provides access for the four domains of 18S rRNA to come together to form a more compact structure (Figure 4). Concurrent with this rRNA collapse is remodeling of rRNA and RNP, which permits formation of the decoding center [38]. Remodeling includes an approximately 90-degree rotation of the 180 Å long helix 44 of the 3′ minor domain and structural rearrangements of the 5′ domain (Figure 4B). Beyer and colleagues may have captured this collapse. Analysis of Miller chromatin spreads of nascent pre-rRNA pol I transcription showed an initial and gradual increase in the size of the SSU terminal knobs on nascent pre-rRNAs, which are expected to be the SSU processome, followed by a compaction [95].

Avoiding the myriad nonfunctional misfolded states is especially acute with LSU rRNA, whose domain structure is complex. Even though the LSU secondary structure has been divided into six domains, its rRNA fold does not have distinct folded domains, as seen with the four SSU 18S rRNA domains. Rather, these six domains intertwine to form a monolithic structure, which was first described in the archaeal LSU [96]. As the 5.8S, ITS2 and 25S RNA sequences emerge from RNA pol I, domains I and II of the 25S RNA fold and interact with 5.8S and ITS2 [85,89,92] to form the solvent exposed back-side of the LSU. As RNA pol I finishes transcription of domain VI, it too folds, but the central domains (III, IV and V) remain disordered and are coated with AFs. Moreover, the 5′ domains are kept physically apart from the 3′ domains by the AFs that coat domains III–V. These domains form the LSU catalytic center, the peptidyl transferase center and the A- and P-sites (domain V), and key structural sites that include the peptide exit tunnel (domains I–V), and the GTPase activating center (domains II and VI). A coordinated dance of entry and exit of different AFs builds these key structures in a hierarchical fashion. For example, once the peptidyl exit tunnel takes shape, a sequential series of AFs (Nog1, Rei1 and Reh1) occupy the tunnel to chaperone folding and prevent its collapse [84,87,91,97]. As the ribosomal precursors migrate to the nucleoplasm and are subsequently exported into the cytoplasm, their function needs to be tested before they are released into the general pool of active ribosomes. The quality-control mechanisms identify and eliminate improperly constructed particles that might inhibit the pool of active subunits. There are quality control tests for both subunits; however, only the SSU quality control will be described here [98,99,100]. SSU quality control involves a pre-40S precursor associated with several AFs and includes ones that sterically block access to the mRNA channel and to the initiator tRNA binding P site. The Eukaryotic Translation Initiation Factor 5B (eIF5B) and the ATPase Fab7 then enables joining this empty SSU to the large 60S subunit. This process tests the function of the GTPase activating center because it requires the GTP hydrolysis of eIF5B. The process also recruits the endonuclease Nob1 to generate the mature 3′ end of the 18S rRNA, releases the remaining AFs and dissociates the two subunits, which signals that this SSU is ready for action. More work is needed to determine which quality control steps are required for proper maturation of all SSUs or only a subset of the population.

## 6. Aging, Nucleolar Pathology and Ribosomopathies

For over a century, it has been recognized that remarkable changes in nucleolar size, shape, and number are a pathological marker for several diseases, including cancer, Alzheimer’s disease and ribosomopathies. Nucleolar size reflects the rate of pre-rRNA transcription, and the progressive aberrant hypertrophy of nucleoli is a useful cytological indicator of tumor malignancy [101,102]. Rapidly proliferating cancer cells require elevated translational capacity and are supported by an increased number of ribosomes per cell. Conversely, recent results suggested that decreased nucleolar size is associated with cellular and organismal longevity in both *Caenorhabditis elegans* and humans [103,104].

Dysfunctions in ribosome biogenesis cause diseases. A common characteristic of immortal cancer cells is the upregulation of ribosome biogenesis, which is needed to ensure that making ribosomes does not limit the rate of cell proliferation. The opposite occurs with aging, where there is a decrease in the cellular rate of ribosome synthesis, which includes reduced expression of both rRNA and r-proteins, and an associated decrease in nucleolar size. This relationship suggests that a slowdown of ribosome biogenesis and protein translation has an impact on increasing life expectancy [105]. Thus, it was not unexpected that epigenetic marks associated with repressing expression of rDNA copies, in particular methylation of CpGs, correlate with age and may function as a biological clock. Recent studies investigated the correlation between age and methylation of CpG islands in the genome [106]. Wang and Lemos found that, in mice, methylation of CpGs with the highest correlation to age was in rDNA and not elsewhere in the genome, with the best model using 72 CpG sites throughout the rDNA gene that included transcribed spacers and rRNAs (18S, 5.8S and 28S). The more intriguing observation was that this “clock” responded to environmental interventions that extend life span (i.e., caloric restriction). Mice on caloric restriction accumulated methylation of rDNA CpGs at a slower rate than those with no feeding restriction, as a result of their aging clock slowing down. More studies on humans and other species are needed to demonstrate the universality and applicability of this biological clock.

Unlike longevity, which is associated with a gradual decrease in the rate of ribosome biogenesis, the premature aging associated with Hutchinson-Gilford progeria syndrome (HGPS) is accompanied by an activation of rDNA transcription and associated nucleolar expansion. Upregulation of ribosome biogenesis and protein synthesis by HGPS-causing progerin expression profoundly exhausts cellular energy metabolism in both non-dividing and dividing cell states. The cellular stress associated with energy depletion contributes to organismal aging in HGPS [107].

For decades it was thought that ribosome synthesis and function were so essential for life and growth that no defect would be tolerated and would lead instead to embryonic lethality. We now know that there are several rare tissue-specific congenital disorders caused by haploinsufficiencies (see [108,109] for more comprehensive reviews). Since ribosomes are universally present and active in all tissues of the body, these findings were somewhat unexpected. The disorders, designated ribosomopathies, arise when there is a loss-of-function mutation in one copy of an r-protein gene or when a factor required for ribosome biogenesis is haploinsufficient. These observations are compatible with embryonic lethality caused by homozygous null alleles of broadly expressed ribosome biogenesis factors [110]. Ribosomopathies exhibit variable penetrance, presumably due to allelic compensation and/or differential tissue expression of variant rDNA genes, which may alter their functions [44,111]. This latter rationale suggests that tissue specific expression of pathological rRNA variants provides one molecular mechanism by which only select tissues are adversely affected, even though the genetic defect occurs in all cells. An alternate view, which is not mutually exclusive, is that some cells are more susceptible to ribosomal stress than others, due to reduced levels of ribosomes, decreased ribosome fidelity, tumor suppressor p53 activation or some combination of these.

Dysfunction of ribosome biogenesis promotes p53 activation, and can differentially impair the translation of highly structured mRNAs with low rates of translational initiation [108]. In healthy cells, the activity of all three RNA polymerases is coordinated in ribosome biogenesis by the synthetic activity of RNA pol I, which results in equimolar levels of rRNA, r-protein-associated enzymes and AFs. For ribosomopathies, this coordination is impaired, resulting in excess 5S ribonucleoprotein complex, which sequesters mouse p53 E3 ubiquitin ligase Mouse Double Minute 2 (MDM2 or HDM2 in humans). As a result, p53, normally made in large quantities and rapidly targeted for ubiquitin-dependent 26S proteasomal degradation, is now stable and its activity results in induction of G1 cell-cycle arrest and apoptosis.

Another molecular consequence of ribosomopathies is a reduced level of functioning ribosomes [108,109]. Those tissues that rely heavily on translation and have greater numbers of ribosomes may be more sensitive to the depletion of functional ribosomes. As a result, mRNAs with a weak affinity for the ribosome will no longer be translated. Also, some ribosomopathies produce modified ribosomes with reduced translational fidelity that can adversely impact cellular function.

Ribosomopathies are of two major classes—those involving inherited bone marrow failure (IBMFSs) and Treacher Collins syndrome (TCS). The first predisposes patients to cancer, and the second, TCS, does not [108]. The IBMFSs include Diamond-Blackfan anemia, Shwachman-Diamond syndrome and Dyskeratosis congenita. These diseases have defects either in r-proteins or in AFs downstream of the initiating and primary control point in ribosome synthesis, which is transcription of the polycistronic 47S pre-rRNA precursor by RNA pol I. A strong selective pressure by IBMFSs to inactivate p53 in the cell, a tumorigenic step, is consistent with an increased susceptibility to cancer. In contrast to the IBMFSs, TCS often downregulates the initiating RNA pol I, which coordinates the activation of RNA pol II and pol III. Due to the coordinated inhibition of the three polymerases used in ribosome biogenesis, most of the other components are downregulated as well. Even though p53 activation leads to cell loss in affected tissues, perhaps the selective pressure to inactivate p53, and thereby promote tumorigenesis, is reduced in TCS due to an overall downregulation of ribosome biogenesis. Clearly, more research is needed to determine why TCS patients are not predisposed to cancer and how differential tissue expression, allelic rDNA variation and rDNA copy number variation contribute to these diseases.

## 7. Concluding Remarks and Future Perspectives

The nucleolus is the most prominent membrane-less nuclear structure of the cell—critically important for the production of ribosomes and essential for protein synthesis in all cells. If the number of functional ribosomes is insufficient, protein translation declines, cell proliferation slows, and cell death may result. Conversely, if the number of functional ribosomes is increased, protein synthesis also increases, cell proliferation accelerates, and cancer may result. Ribosome biogenesis is highly responsive to changes in environmental conditions that stress the cell, such as metabolic stress, DNA damage, and proteotoxic stress induced by the inhibition of ubiquitin-proteasome function. When rDNA transcription and ribosome assembly are disturbed, the nucleolus quickly becomes the central hub that coordinates the stress response. The inhibition of ribosome production results in a cascade of nucleolus-mediated molecular events that either maintain homeostasis or initiate severe responses such as cell cycle arrest and apoptosis. Attention to the general mechanisms that regulate ribosome production and translation under different stress conditions may reveal new strategies with the potential to target diseases characterized by pathological cell proliferation, such as cancer.

The newly emerging principles of liquid-liquid phase separation offer a fresh perspective and new approaches with which to unravel the role of pre-rRNA and other non-coding RNAs in the formation and regulation of distinct functional phases within the nucleolus. How exactly does the dynamic interplay between nascent rRNA transcription and the processing machinery phase separate from numerous intermediates in the assembly pipeline of pre-ribosomal subunits?

Given current advances in genome-wide sequencing, we look forward to a full sequence and map of the entire NORs, including regions around centromeres and telomeres on each NOR-bearing acrocentric chromosome. Analysis of these sequences (such as the recently characterized DJ region [42]) will shed light on the role of NOR-containing acrocentric chromosome ends in the higher-order spatial organization of the genome and their rearrangement during formation of the nucleolus, and later during formation of larger mature nucleoli through fusion events. How each NOR contributes to the topological organization of the genome around the nucleolus will be revealed as well.

Continued deep sequencing will uncover new mutations in alleles for ribosomopathies and cancer-associated ribosomal gene mutations. Characterization of their functional effects will further impact our understanding of the relevant pathologies, potentially leading to novel therapeutic strategies. Future studies will undoubtedly provide fresh insights into the functional complexity of the nucleolus, its roles in maintaining cell homeostasis and its involvement in life-threatening pathological conditions and aging.

## Abbreviations

AF	assembly factor
BMC	biomolecular condensate
CPK	central pseudoknot
DFC	dense fibrillar component
ETS	external-transcribed spacer
FC	fibrillar left
GC	granular component
IDR	intrinsically disordered region
IGS	intergenic spacer
ITS	internal-transcribed spacer
LSU	Large subunit
NOR	nucleolus organizer region
NoRC	nucleolar repressive complex
pRNA	promoter-associated RNA
RNP	ribonucleoprotein
RNA pol	RNA polymerase
r-protein	ribosomal protein
rRNA	ribosomal RNA
snoRNP	small nucleolar ribonucleoprotein
SSU processome	Small Subunit processome

## References

[1] Boeynaems S., Alberti S., Fawzi N.L., Mittag T., Polymenidou M., Rousseau F., Schymkowitz J., Shorter J., Wolozin B., Van Den Bosch L. (2018). Protein Phase Separation: A New Phase in Cell Biology. Trends Cell Biol..

[2] Gomes E., Shorter J. (2019). The molecular language of membraneless organelles. J. Biol. Chem..

[3] Sawyer I.A., Bartek J., Dundr M. (2019). Phase separated microenvironments inside the cell nucleus are linked to disease and regulate epigenetic state, transcription and RNA processing. Semin. Cell Dev. Biol..

[4] Wang Z., Zhang H. (2019). Phase Separation, Transition, and Autophagic Degradation of Proteins in Development and Pathogenesis. Trends Cell Biol..

[5] Woodruff J.B., Hyman A.A., Boke E. (2018). Organization and Function of Non-dynamic Biomolecular Condensates. Trends Biochem. Sci..

[6] Sawyer I.A., Dundr M. (2016). Nuclear bodies: Built to boost. J. Cell Biol..

[7] Stroberg W., Schnell S. (2018). Do Cellular Condensates Accelerate Biochemical Reactions? Lessons from Microdroplet Chemistry. Biophys. J..

[8] Brangwynne C.P., Mitchison T.J., Hyman A.A. (2011). Active liquid-like behavior of nucleoli determines their size and shape in Xenopus laevis oocytes. Proc. Natl. Acad. Sci. USA.

[9] Feric M., Vaidya N., Harmon T.S., Mitrea D.M., Zhu L., Richardson T.M., Kriwacki R.W., Pappu R.V., Brangwynne C.P. (2016). Coexisting Liquid Phases Underlie Nucleolar Subcompartments. Cell.

[10] Mitrea D.M., Cika J.A., Guy C.S., Ban D., Banerjee P.R., Stanley C.B., Nourse A., Deniz A.A., Kriwacki R.W. (2016). Nucleophosmin integrates within the nucleolus via multi-modal interactions with proteins displaying R-rich linear motifs and rRNA. eLife.

[11] Fay M.M., Anderson P.J. (2018). The Role of RNA in Biological Phase Separations. J. Mol. Biol..

[12] Lewis J.D., Tollervey D. (2000). Like attracts like: Getting RNA processing together in the nucleus. Science.

[13] Henderson A.S., Warburton D., Atwood K.C. (1972). Location of ribosomal DNA in the human chromosome complement. Proc. Natl. Acad. Sci. USA.

[14] Kalmarova M., Smirnov E., Masata M., Koberna K., Ligasova A., Popov A., Raska I. (2007). Positioning of NORs and NOR-bearing chromosomes in relation to nucleoli. J. Struct. Biol..

[15] Parada L.A., McQueen P.G., Munson P.J., Misteli T. (2002). Conservation of relative chromosome positioning in normal and cancer cells. Curr. Biol..

[16] Mao Y.S., Sunwoo H., Zhang B., Spector D.L. (2011). Direct visualization of the co-transcriptional assembly of a nuclear body by noncoding RNAs. Nat. Cell Biol..

[17] Shevtsov S.P., Dundr M. (2011). Nucleation of nuclear bodies by RNA. Nat. Cell Biol..

[18] Chujo T., Hirose T. (2017). Nuclear Bodies Built on Architectural Long Noncoding RNAs: Unifying Principles of Their Construction and Function. Mol. Cells.

[19] Chujo T., Yamazaki T., Kawaguchi T., Kurosaka S., Takumi T., Nakagawa S., Hirose T. (2017). Unusual semi-extractability as a hallmark of nuclear body-associated architectural noncoding RNAs. EMBO J..

[20] Michieletto D., Gilbert N. (2019). Role of nuclear RNA in regulating chromatin structure and transcription. Curr. Opin. Cell Biol..

[21] Turoverov K.K., Kuznetsova I.M., Fonin A.V., Darling A.L., Zaslavsky B.Y., Uversky V.N. (2019). Stochasticity of Biological Soft Matter: Emerging Concepts in Intrinsically Disordered Proteins and Biological Phase Separation. Trends Biochem. Sci..

[22] Uversky V.N. (2017). Intrinsically disordered proteins in overcrowded milieu: Membrane-less organelles, phase separation, and intrinsic disorder. Curr. Opin. Struct. Biol..

[23] Nguemaha V., Zhou H.X. (2018). Liquid-Liquid Phase Separation of Patchy Particles Illuminates Diverse Effects of Regulatory Components on Protein Droplet Formation. Sci. Rep..

[24] Wei M.T., Elbaum-Garfinkle S., Holehouse A.S., Chen C.C., Feric M., Arnold C.B., Priestley R.D., Pappu R.V., Brangwynne C.P. (2017). Phase behaviour of disordered proteins underlying low density and high permeability of liquid organelles. Nat. Chem..

[25] O’Neil D., Glowatz H., Schlumpberger M. (2013). Ribosomal RNA depletion for efficient use of RNA-seq capacity. Curr. Protoc. Mol. Biol..

[26] Albert B., Perez-Fernandez J., Leger-Silvestre I., Gadal O. (2012). Regulation of ribosomal RNA production by RNA polymerase I: Does elongation come first?. Genet. Res. Int..

[27] Miller O.L., Beatty B.R. (1969). Visualization of nucleolar genes. Science.

[28] Jackson D.A., Iborra F.J., Manders E.M., Cook P.R. (1998). Numbers and organization of RNA polymerases, nascent transcripts, and transcription units in HeLa nuclei. Mol. Biol. Cell.

[29] Denissov S., Lessard F., Mayer C., Stefanovsky V., van Driel M., Grummt I., Moss T., Stunnenberg H.G. (2011). A model for the topology of active ribosomal RNA genes. EMBO Rep..

[30] Dundr M. (2012). Nuclear bodies: Multifunctional companions of the genome. Curr. Opin. Cell Biol..

[31] Koberna K., Malinsky J., Pliss A., Masata M., Vecerova J., Fialova M., Bednar J., Raska I. (2002). Ribosomal genes in focus: New transcripts label the dense fibrillar components and form clusters indicative of “Christmas trees” in situ. J. Cell Biol..

[32] Schofer C., Weipoltshammer K. (2018). Nucleolus and chromatin. Histochem Cell Biol.

[33] Pelletier J., Thomas G., Volarevic S. (2018). Ribosome biogenesis in cancer: New players and therapeutic avenues. Nat. Rev. Cancer.

[34] Stults D.M., Killen M.W., Pierce H.H., Pierce A.J. (2008). Genomic architecture and inheritance of human ribosomal RNA gene clusters. Genome Res..

[35] Gibbons J.G., Branco A.T., Godinho S.A., Yu S., Lemos B. (2015). Concerted copy number variation balances ribosomal DNA dosage in human and mouse genomes. Proc. Natl. Acad. Sci. USA.

[36] Fatica A., Tollervey D. (2002). Making ribosomes. Curr. Opin. Cell Biol..

[37] Bassler J., Hurt E. (2019). Eukaryotic Ribosome Assembly. Annu. Rev. Biochem..

[38] Klinge S., Woolford J.L. (2019). Ribosome assembly coming into focus. Nat. Rev. Mol. Cell Biol..

[39] Khatter H., Myasnikov A.G., Natchiar S.K., Klaholz B.P. (2015). Structure of the human 80S ribosome. Nature.

[40] Melnikov S., Ben-Shem A., Garreau de Loubresse N., Jenner L., Yusupova G., Yusupov M. (2012). One core, two shells: Bacterial and eukaryotic ribosomes. Nat. Struct. Mol. Biol..

[41] Frottin F., Schueder F., Tiwary S., Gupta R., Korner R., Schlichthaerle T., Cox J., Jungmann R., Hartl F.U., Hipp M.S. (2019). The nucleolus functions as a phase-separated protein quality control compartment. Science.

[42] Mangan H., Gailin M.O., McStay B. (2017). Integrating the genomic architecture of human nucleolar organizer regions with the biophysical properties of nucleoli. FEBS J..

[43] Van Sluis M., McStay B. (2017). Nucleolar reorganization in response to rDNA damage. Curr. Opin. Cell Biol..

[44] Parks M.M., Kurylo C.M., Dass R.A., Bojmar L., Lyden D., Vincent C.T., Blanchard S.C. (2018). Variant ribosomal RNA alleles are conserved and exhibit tissue-specific expression. Sci. Adv..

[45] McStay B. (2016). Nucleolar organizer regions: Genomic ‘dark matter’ requiring illumination. Genes Dev..

[46] Nemeth A., Langst G. (2011). Genome organization in and around the nucleolus. Trends Genet..

[47] Caburet S., Conti C., Schurra C., Lebofsky R., Edelstein S.J., Bensimon A. (2005). Human ribosomal RNA gene arrays display a broad range of palindromic structures. Genome Res..

[48] Wang M., Tao X., Jacob M.D., Bennett C.A., Ho J.J.D., Gonzalgo M.L., Audas T.E., Lee S. (2018). Stress-Induced Low Complexity RNA Activates Physiological Amyloidogenesis. Cell Rep..

[49] Dillinger S., Straub T., Nemeth A. (2017). Nucleolus association of chromosomal domains is largely maintained in cellular senescence despite massive nuclear reorganisation. PLoS ONE.

[50] Iarovaia O.V., Minina E.P., Sheval E.V., Onichtchouk D., Dokudovskaya S., Razin S.V., Vassetzky Y.S. (2019). Nucleolus: A Central Hub for Nuclear Functions. Trends Cell Biol..

[51] McStay B., Grummt I. (2008). The epigenetics of rRNA genes: From molecular to chromosome biology. Annu. Rev. Cell Dev. Biol..

[52] Conconi A., Widmer R.M., Koller T., Sogo J.M. (1989). Two different chromatin structures coexist in ribosomal RNA genes throughout the cell cycle. Cell.

[53] Santoro R., Li J., Grummt I. (2002). The nucleolar remodeling complex NoRC mediates heterochromatin formation and silencing of ribosomal gene transcription. Nat. Genet..

[54] Zentner G.E., Saiakhova A., Manaenkov P., Adams M.D., Scacheri P.C. (2011). Integrative genomic analysis of human ribosomal DNA. Nucleic Acids Res..

[55] Herdman C., Mars J.C., Stefanovsky V.Y., Tremblay M.G., Sabourin-Felix M., Lindsay H., Robinson M.D., Moss T. (2017). A unique enhancer boundary complex on the mouse ribosomal RNA genes persists after loss of Rrn3 or UBF and the inactivation of RNA polymerase I transcription. PLoS Genet..

[56] Nguyen le X.T., Raval A., Garcia J.S., Mitchell B.S. (2015). Regulation of ribosomal gene expression in cancer. J. Cell Physiol..

[57] Wang M., Lemos B. (2017). Ribosomal DNA copy number amplification and loss in human cancers is linked to tumor genetic context, nucleolus activity, and proliferation. PLoS Genet..

[58] Xu B., Li H., Perry J.M., Singh V.P., Unruh J., Yu Z., Zakari M., McDowell W., Li L., Gerton J.L. (2017). Ribosomal DNA copy number loss and sequence variation in cancer. PLoS Genet..

[59] Sanij E., Poortinga G., Sharkey K., Hung S., Holloway T.P., Quin J., Robb E., Wong L.H., Thomas W.G., Stefanovsky V. (2008). UBF levels determine the number of active ribosomal RNA genes in mammals. J. Cell Biol..

[60] Lawrence R.J., Earley K., Pontes O., Silva M., Chen Z.J., Neves N., Viegas W., Pikaard C.S. (2004). A concerted DNA methylation/histone methylation switch regulates rRNA gene dosage control and nucleolar dominance. Mol. Cell.

[61] Penzo M., Casoli L., Pollutri D., Sicuro L., Ceccarelli C., Santini D., Taffurelli M., Govoni M., Brina D., Trere D. (2015). JHDM1B expression regulates ribosome biogenesis and cancer cell growth in a p53 dependent manner. Int. J. Cancer.

[62] Leone S., Bar D., Slabber C.F., Dalcher D., Santoro R. (2017). The RNA helicase DHX9 establishes nucleolar heterochromatin, and this activity is required for embryonic stem cell differentiation. EMBO Rep..

[63] Santoro R., Schmitz K.M., Sandoval J., Grummt I. (2010). Intergenic transcripts originating from a subclass of ribosomal DNA repeats silence ribosomal RNA genes in trans. EMBO Rep..

[64] Guetg C., Lienemann P., Sirri V., Grummt I., Hernandez-Verdun D., Hottiger M.O., Fussenegger M., Santoro R. (2010). The NoRC complex mediates the heterochromatin formation and stability of silent rRNA genes and centromeric repeats. EMBO J..

[65] Guetg C., Scheifele F., Rosenthal F., Hottiger M.O., Santoro R. (2012). Inheritance of silent rDNA chromatin is mediated by PARP1 via noncoding RNA. Mol. Cell.

[66] Zhou Y., Grummt I. (2005). The PHD finger/bromodomain of NoRC interacts with acetylated histone H4K16 and is sufficient for rDNA silencing. Curr. Biol..

[67] Gagnon-Kugler T., Langlois F., Stefanovsky V., Lessard F., Moss T. (2009). Loss of human ribosomal gene CpG methylation enhances cryptic RNA polymerase II transcription and disrupts ribosomal RNA processing. Mol. Cell.

[68] Mayer C., Schmitz K.M., Li J., Grummt I., Santoro R. (2006). Intergenic transcripts regulate the epigenetic state of rRNA genes. Mol. Cell.

[69] Tsekrekou M., Stratigi K., Chatzinikolaou G. (2017). The Nucleolus: In Genome Maintenance and Repair. Int. J. Mol. Sci.

[70] Lindstrom M.S., Jurada D., Bursac S., Orsolic I., Bartek J., Volarevic S. (2018). Nucleolus as an emerging hub in maintenance of genome stability and cancer pathogenesis. Oncogene.

[71] Ganley A.R., Kobayashi T. (2014). Ribosomal DNA and cellular senescence: New evidence supporting the connection between rDNA and aging. FEMS Yeast Res..

[72] Killen M.W., Stults D.M., Adachi N., Hanakahi L., Pierce A.J. (2009). Loss of Bloom syndrome protein destabilizes human gene cluster architecture. Hum. Mol. Genet..

[73] Srivastava R., Srivastava R., Ahn S.H. (2016). The Epigenetic Pathways to Ribosomal DNA Silencing. Microbiol. Mol. Biol. Rev..

[74] Harding S.M., Boiarsky J.A., Greenberg R.A. (2015). ATM Dependent Silencing Links Nucleolar Chromatin Reorganization to DNA Damage Recognition. Cell Rep..

[75] Kruhlak M., Crouch E.E., Orlov M., Montano C., Gorski S.A., Nussenzweig A., Misteli T., Phair R.D., Casellas R. (2007). The ATM repair pathway inhibits RNA polymerase I transcription in response to chromosome breaks. Nature.

[76] Larsen D.H., Hari F., Clapperton J.A., Gwerder M., Gutsche K., Altmeyer M., Jungmichel S., Toledo L.I., Fink D., Rask M.B. (2014). The NBS1-Treacle complex controls ribosomal RNA transcription in response to DNA damage. Nat. Cell Biol..

[77] Van Sluis M., McStay B. (2015). A localized nucleolar DNA damage response facilitates recruitment of the homology-directed repair machinery independent of cell cycle stage. Genes Dev..

[78] Warmerdam D.O., van den Berg J., Medema R.H. (2016). Breaks in the 45S rDNA Lead to Recombination-Mediated Loss of Repeats. Cell Rep..

[79] Korsholm L.M., Gal Z., Lin L., Quevedo O., Ahmad D.A., Dulina E., Luo Y., Bartek J., Larsen D.H. (2019). Double-strand breaks in ribosomal RNA genes activate a distinct signaling and chromatin response to facilitate nucleolar restructuring and repair. Nucleic Acids Res..

[80] Ben-Shem A., Garreau de Loubresse N., Melnikov S., Jenner L., Yusupova G., Yusupov M. (2011). The structure of the eukaryotic ribosome at 3.0 A resolution. Science.

[81] Barandun J., Chaker-Margot M., Hunziker M., Molloy K.R., Chait B.T., Klinge S. (2017). The complete structure of the small-subunit processome. Nat. Struct. Mol. Biol..

[82] Barrio-Garcia C., Thoms M., Flemming D., Kater L., Berninghausen O., Bassler J., Beckmann R., Hurt E. (2016). Architecture of the Rix1-Rea1 checkpoint machinery during pre-60S-ribosome remodeling. Nat. Struct. Mol. Biol..

[83] Cheng J., Kellner N., Berninghausen O., Hurt E., Beckmann R. (2017). 3.2-A-resolution structure of the 90S preribosome before A1 pre-rRNA cleavage. Nat. Struct. Mol. Biol.

[84] Greber B.J., Gerhardy S., Leitner A., Leibundgut M., Salem M., Boehringer D., Leulliot N., Aebersold R., Panse V.G., Ban N. (2016). Insertion of the Biogenesis Factor Rei1 Probes the Ribosomal Tunnel during 60S Maturation. Cell.

[85] Kater L., Thoms M., Barrio-Garcia C., Cheng J., Ismail S., Ahmed Y.L., Bange G., Kressler D., Berninghausen O., Sinning I. (2017). Visualizing the Assembly Pathway of Nucleolar Pre-60S Ribosomes. Cell.

[86] Kornprobst M., Turk M., Kellner N., Cheng J., Flemming D., Kos-Braun I., Kos M., Thoms M., Berninghausen O., Beckmann R. (2016). Architecture of the 90S Pre-ribosome: A Structural View on the Birth of the Eukaryotic Ribosome. Cell.

[87] Ma C., Wu S., Li N., Chen Y., Yan K., Li Z., Zheng L., Lei J., Woolford J.L., Gao N. (2017). Structural snapshot of cytoplasmic pre-60S ribosomal particles bound by Nmd3, Lsg1, Tif6 and Reh. Nat. Struct. Mol. Biol..

[88] Malyutin A.G., Musalgaonkar S., Patchett S., Frank J., Johnson A.W. (2017). Nmd3 is a structural mimic of eIF5A, and activates the cpGTPase Lsg1 during 60S ribosome biogenesis. EMBO J..

[89] Sanghai Z.A., Miller L., Molloy K.R., Barandun J., Hunziker M., Chaker-Margot M., Wang J., Chait B.T., Klinge S. (2018). Modular assembly of the nucleolar pre-60S ribosomal subunit. Nature.

[90] Sun Q., Zhu X., Qi J., An W., Lan P., Tan D., Chen R., Wang B., Zheng S., Zhang C. (2017). Molecular architecture of the 90S small subunit pre-ribosome. eLife.

[91] Wu S., Tutuncuoglu B., Yan K., Brown H., Zhang Y., Tan D., Gamalinda M., Yuan Y., Li Z., Jakovljevic J. (2016). Diverse roles of assembly factors revealed by structures of late nuclear pre-60S ribosomes. Nature.

[92] Zhou D., Zhu X., Zheng S., Tan D., Dong M.Q., Ye K. (2019). Cryo-EM structure of an early precursor of large ribosomal subunit reveals a half-assembled intermediate. Protein Cell.

[93] Sa-Moura B., Kornprobst M., Kharde S., Ahmed Y.L., Stier G., Kunze R., Sinning I., Hurt E. (2017). Mpp10 represents a platform for the interaction of multiple factors within the 90S pre-ribosome. PLoS ONE.

[94] Sardana R., Liu X., Granneman S., Zhu J., Gill M., Papoulas O., Marcotte E.M., Tollervey D., Correll C.C., Johnson A.W. (2015). The DEAH-box helicase Dhr1 dissociates U3 from the pre-rRNA to promote formation of the central pseudoknot. PLoS Biol..

[95] Osheim Y.N., French S.L., Keck K.M., Champion E.A., Spasov K., Dragon F., Baserga S.J., Beyer A.L. (2004). Pre-18S ribosomal RNA is structurally compacted into the SSU processome prior to being cleaved from nascent transcripts in Saccharomyces cerevisiae. Mol. Cell.

[96] Ban N., Nissen P., Hansen J., Moore P.B., Steitz T.A. (2000). The complete atomic structure of the large ribosomal subunit at 2.4 A resolution. Science.

[97] Greber B.J., Boehringer D., Montellese C., Ban N. (2012). Cryo-EM structures of Arx1 and maturation factors Rei1 and Jjj1 bound to the 60S ribosomal subunit. Nat. Struct. Mol. Biol..

[98] Ghalei H., Trepreau J., Collins J.C., Bhaskaran H., Strunk B.S., Karbstein K. (2017). The ATPase Fap7 Tests the Ability to Carry Out Translocation-like Conformational Changes and Releases Dim1 during 40S Ribosome Maturation. Mol. Cell.

[99] Lebaron S., Schneider C., van Nues R.W., Swiatkowska A., Walsh D., Bottcher B., Granneman S., Watkins N.J., Tollervey D. (2012). Proofreading of pre-40S ribosome maturation by a translation initiation factor and 60S subunits. Nat. Struct. Mol. Biol..

[100] Strunk B.S., Novak M.N., Young C.L., Karbstein K. (2012). A translation-like cycle is a quality control checkpoint for maturing 40S ribosome subunits. Cell.

[101] Derenzini M., Montanaro L., Trere D. (2017). Ribosome biogenesis and cancer. Acta Histochem..

[102] Derenzini M., Trere D., Pession A., Montanaro L., Sirri V., Ochs R.L. (1998). Nucleolar function and size in cancer cells. Am. J. Pathol..

[103] Tiku V., Antebi A. (2018). Nucleolar Function in Lifespan Regulation. Trends Cell Biol..

[104] Uppaluri S., Weber S.C., Brangwynne C.P. (2016). Hierarchical Size Scaling during Multicellular Growth and Development. Cell Rep..

[105] Tiku V., Jain C., Raz Y., Nakamura S., Heestand B., Liu W., Spath M., Suchiman H.E.D., Muller R.U., Slagboom P.E. (2017). Small nucleoli are a cellular hallmark of longevity. Nat. Commun..

[106] Wang M., Lemos B. (2019). Ribosomal DNA harbors an evolutionarily conserved clock of biological aging. Genome Res..

[107] Buchwalter A., Hetzer M.W. (2017). Nucleolar expansion and elevated protein translation in premature aging. Nat. Commun..

[108] Aspesi A., Ellis S.R. (2019). Rare ribosomopathies: Insights into mechanisms of cancer. Nat. Rev. Cancer.

[109] Mills E.W., Green R. (2017). Ribosomopathies: There’s strength in numbers. Science.

[110] Freed E.F., Bleichert F., Dutca L.M., Baserga S.J. (2010). When ribosomes go bad: Diseases of ribosome biogenesis. Mol. Biosyst..

[111] Kurylo C.M., Parks M.M., Juette M.F., Zinshteyn B., Altman R.B., Thibado J.K., Vincent C.T., Blanchard S.C. (2018). Endogenous rRNA Sequence Variation Can Regulate Stress Response Gene Expression and Phenotype. Cell Rep..

